# Fabrication of ZrO_2_ Armor Ceramics by 3D Printing Accompanied with Microwave Sintering

**DOI:** 10.3390/ma17246034

**Published:** 2024-12-10

**Authors:** Zhengang Liang, Dongjiang Zhang, Xin Chen, Chunxu Pang, Xuncheng Guo, Yanfei Feng, Xiqing Xu

**Affiliations:** 1 School of Equipment Engineering, Shenyang Ligong University, Shenyang 110159, China; 2 Xi’an Modern Control Technology Research Institute, Xi’an 710065, China; 3 School of Materials Science and Engineering, Chang’an University, Xi’an 710061, China; 4 State Key Laboratory of New Textile Materials and Advanced Processing Technologies, College of Textile Science and Engineering, Wuhan Textile University, Wuhan 430200, China

**Keywords:** 3D printing, ZrO_2_ ceramics, photosensitive resin, microwave sintering, microstructure

## Abstract

Ceramic armor protection with complex shapes is limited by the difficult molding or machining processing, and 3D printing technology provides a feasible method for complex-shaped ceramics. In this study, ZrO_2_ ceramics were manufactured by 3D printing accompanied with microwave sintering. In 3D printing, the formula of photosensitive resin was optimized by controlling the content of polyurethane acrylic (PUA) as oligomer, and the photosensitive resin with 50% PUA showed excellent curing performance with a small volume shrinkage of 4.05%, media viscosity of 550 mPa·s, and low critical exposure of 20 mJ/cm^2^. Compared to conventional sintering, microwave sintering was beneficial to dense microstructures with fine grain size, and microwave sintering at 1500 °C was confirmed as an optimized sintering process for the 3D-printed ZrO_2_ ceramics, and the obtained ceramics showed a relative density of 98.2% and mean grain size of 2.1 μm. The PUA content further affected the microstructure and mechanical property of the ZrO_2_ ceramics. The sample with 10%~40% PUA showed some pores due to the low viscosity and large volume shrinkage of photosensitive resins, and the sample with 60% PUA exhibited an inhomogeneous microstructure with agglomeration, attributed to the high viscosity of photosensitive resins. Finally, the ZrO_2_ ceramics via 3D printing with 50% PUA showed superior mechanical properties, whose Vickers hardness was 3.4 GPa, fracture toughness was 7.4 MPa·m^1/2^, flexure strength was 1038 MPa, and dynamic strength at 1200 s^−1^ was 4.9 GPa, conducive to the material’s employment as armor protection ceramics.

## 1. Introduction

Ceramic materials are characterized by their high hardness, high-temperature resistance, high strength, and excellent chemical durability, and they have become key armor protection materials [1,2,3]. The extreme high hardness and strength [4] of ceramics are conducive to resisting the penetration of high-speed armor-piercing bullets. Their superior heat resistance [5] effectively resists the penetration of high-temperature metal jets. Their chemical durability [6] ensures the stability of structure and performance in extreme environments. However, brittleness is one of the fatal weaknesses of ceramics [7,8], which leads to low strength before fragmentation under projectile impact, and difficulty in machining processing. With the rapid development of armor protection systems, their shape has become more and more complex. The processing of high-hardness ceramics requires expensive grinding tools [9], leading to the high cost and low profit of ceramic armor materials. Mold forming is another method of manufacturing ceramics with designed shapes, but the employment of molds extends the production cycle and limits the dimensional accuracy.

Compared with machining or mold forming, 3D printing [10,11,12] is an optimal technology for the molding of complex-shaped ceramic armor materials, by virtue of its high precision, high molding quality, and high molding efficiency. At present, various 3D printing technologies have been used for ceramic materials, including direct writing, fused deposition modeling, laser selective sintering, and so on, among which digital light processing (DLP) 3D printing [13,14] is an optimized technology for complex-shaped ceramic armor, due to the rapid molding and fine accuracy.

In DLP 3D printing, liquid photosensitive resin [15,16] doped with ceramic powders was treated under ultraviolet (UV) light, and the photopolymerization of resin bonds the ceramic powders into volumes with suitable strength and smoothness. Therefore, the quality of printed ceramic parts is highly dependent on the photosensitive resin. Oligomer, one of the essential components of photosensitive resins [17], is an organic matter with relatively low molecular weight and photo curing reactive groups. PUA is a superior oligomer with good flexibility, high wear resistance, strong adhesion, high tear strength, and optical properties [18,19]; however, it is limited by its slow light curing speed and high viscosity. Therefore, PUA is generally combined with low-viscous monomers [20], and the detailed formula is extremely important to the printing performance of photosensitive resins and properties of printed ceramic samples.

ZrO_2_ ceramic is one of the most toughness ceramics, making it widely employed in aerospace, metallurgy, biomedicine, and armor protection [21,22,23]. For ZrO_2_ ceramics, the sintering from powders into high-density bulks requires high temperature, which not only consumes huge energy, but also induces obvious grain growth and counts against the mechanical property [24,25]. To avoid obvious grain growth in sintering, varieties of technologies have been conducted, including spark plasma sintering [26,27], hot pressing [28], hot isostatic pressing [29], and so on. However, those methods rely on the assistance of pressure in sintering, which is inapplicable to ceramics with complex shapes [30]. Microwave sintering [31,32,33] has a fast heating rate, high energy utilization efficiency, simplicity, and is pollution-free, which can improve the properties of sintered materials, making it a suitable sintering method for ZrO_2_ ceramics with maintained shapes.

Herein, ZrO_2_ armor ceramics were fabricated by DLP 3D printing along with microwave sintering. In 3D printing, PUA was employed as oligomer mixed with monomers, and the effects of PUA contents on the printing performance were investigated. The ceramics after printing were sintered by microwave sintering or conventional sintering, and the microstructures at different temperatures were investigated to determine the optimum sintering technique. Furthermore, the mechanical properties of ZrO_2_ armor ceramics dependent on the PUA contents were explored.

## 2. Materials and Methods

### 2.1. Photosensitive Slurry

To form the photosensitive resin, polyurethane acrylate (PUA) was employed as oligomer, dipropylene glycol diacrylate (DPGDA), 16-hexanediol diacrylate (HDDA), and trimethylolpropane triacrylate (TMPTA) in a mass ration of 1:1:1 were used as mixed monomers, and 2,4,6-trimethylbenzoyl ethoxy phenyl phosphine oxide (TPO-L) was employed as photoinitiator. The monomer and oligomer were mixed in different ratios, with the content of oligomer being 10 wt% to 60 wt%, respectively. After mechanical agitation for 2 h, the photosensitive resin was placed in quiet until the full disappearance of bubbles. The oligomer, monomer, and photoinitiator with analytical purity in this work were produced by Shanghai Yinchang Co., Ltd., Shanghai, China.

**Figure 1:**
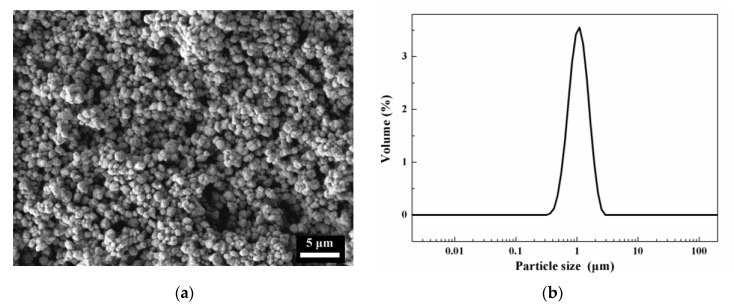
(**a**) SEM image and (**b**) particle size distribution of the ZrO_2_ powders.

The solid phase employed in the photosensitive slurry was ZrO_2_ powder stabilized by 3 mol% Y_2_O_3_ (3YSZ, analytical pure, purity > 99.99%), which was purchased from Tosoh Co., Ltd., Japan. The detailed microstructure and particle size distribution are displayed in Figure 1, exhibiting a uniform distribution of low-aggregated ZrO_2_ powders with an average size of about 1 μm. The pre-mixed photosensitive resin and the ZrO_2_ powders were ball milled for 6 h, after which filtering and vacuum defoaming were conducted to obtain a photocurable ceramic slurry in a solid loading of 56 vol%.

### 2.2. 3D Printing and Sintering

**Figure 2:**
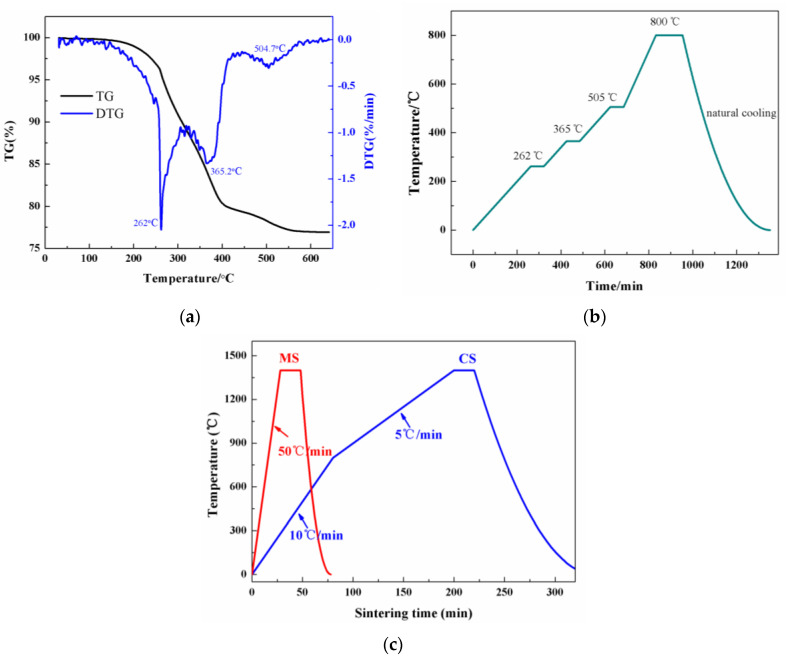
(**a**) TG-DTG curves of the green body manufactured by 3D printing; (**b**) the degreasing procedure of ceramic green bodies; (**c**) the sintering procedure of ceramics in conventional sintering (CS) and microwave sintering (MS).

3D printing was performed on the photosensitive ceramic slurry with PUA contents of 10 wt% to 60 wt% to mold bulk samples, using a DLP 3D printer (Autocera-M, Beijing Shiwei Technology, Beijing, China) according to digital models. After molding in the printer, the excess slurry was washed in an ultrasonic cleaner accompanied with oven drying. The degreasing were performed in a muffle furnace under a detailed temperature program based on the thermogravimetric analysis-differential thermogravimetric analysis (TG-DTG) curve in Figure 2a. The sample exhibited significant mass loss at 262 °C, 365 °C, and 505 °C, respectively. After 600 °C, the TG curve tends to stabilize, suggesting that the organics in the ceramic slurry were thermally decomposed completely. Therefore, the detailed degreasing procedure was determined as shown in Figure 2b. During degreasing, the temperature was held at 262 °C, 365 °C, and 505 °C for 1 h, respectively, in order to guarantee slow degreasing without damage to the ceramic bodies by thermal stress. After holding at 505 °C, the sample was heated to 800 °C and maintained for 2 h before natural cooling.

To transform the porous green body into dense structures, two kinds of sintering were employed, i.e., conventional sintering (CS) and microwave sintering (MS). Conventional sintering was conducted in a muffle furnace, and microwave sintering was performed using a microwave furnace (1.1-kW, 2.45-GHz, Bosch, Gerlingen, Germany), both of which were held at 1200~1600 °C for 20 min before natural cooling, and the detailed procedure of sintering is exhibited in Figure 2c.

### 2.3. Characterization and Testing

A viscometer (THS-NDJ-5S, Shenzhen, China) was performed to measure the viscosity of ceramic slurry and photosensitive resin under 50 r/min at 25 °C. A vernier caliper was employed to measure the dimension of the green body, and the volume shrinkage during 3D printing was determined based on the theoretical dimensions digital model. The curing depth after exposure was tested using a spiral micrometer, based on the average value of five measurements.

The critical exposure along with critical transmission depth of the different photosensitive resins was calculated through the Beer Lambert formula [34,35]:

(1) $$C_{d}=D_{P}\ln\left(E/E_{c}\right),$$

where the *C_d_* is the curing depth (μm), *D_p_* is the depth of penetration (μm), *E* indicates the actual exposure energy fluence (mJ/cm^2^), and *E_c_* is the critical energy (mJ/cm^2^), which represents the minimum energy required for the curing of the slurry. The value of *C_d_* was calculated and plotted as the function of ln⁡E, and the values of *D_p_* and *E_c_* can be obtained based on the slope and intercept.

The TG-DTG curves were measured on the green body manufactured by 3D printing, using a thermal analyzer (TG209F1, NETZSCH, Selb, Germany), in which the heating rate was 5 °C/min. The microstructure of the ZrO_2_ ceramics with different photosensitive resins after sintering was analyzed by a scanning electron microscope (SEM, JSM-5600LV, JEOL, Tokyo, Japan). The grain sizes in the sintered ceramics were tested by the software Nano Measurer 1.2. The phase composition of the samples was investigated using X-ray diffraction (XRD, Bruker D8 Davinci, Karlsruhe, Germany). The bulk density and apparent porosity were tested by the Archimedes’ method via ASTM C373, and the relative density was obtained as the true density of t-ZrO_2_ was 6.10 g/cm^3^.

The bending strengths of the sintered specimens at room temperature were tested, during which an electronic universal testing machine (Instron5500R, Norwood, MA, USA) was employed. The sintered bars were of dimensions of 1.5 × 2 × 20 mm, with the supporting span of 15 mm and loading rate of 1 mm/min. The flexure strength (σ) was determined based on the equation [36]

(2) $$\sigma =\frac{3\mathrm{PL}}{2bd^{2}}.$$

where P was the load at which the bars broke down, L was the support span, b was the specimen width, and d was the specimen thickness. Each measurement was tested on 8 specimens, and the mean values of these measurements were taken as the three-point bending values.

The Vickers hardness (H_V_) was measured using a microhardness tester (HXD-1000TM, Shanghai, China) under 9.8 N for 10 s. Based on the diagonal length of indentation, the H_V_ was evaluated via the equation [37]

(3) $$H_{V}=1.854\frac{P}{d^{2}}$$

where P represented the load and d expressed the mean value of the diagonal length. Each value of H_V_ took an average value of 20 indentations.

The fracture toughness (K_IC_) was tested via the single-edge notched beam method (SENB) with a notch depth of 2.5 mm. The dimensions of the testing bars were 30 × 6 × 4 mm in length, thickness, and width, with a notch depth of 2.5 mm, and the supporting span in three-point bending was 20 mm. The fracture toughness through SENB method could be expressed by [38]

(4) $$K_{\mathrm{IC}}=Y\frac{3\mathrm{PL}}{2bh^{2}}\sqrt{a}.$$

(5) $$Y=1.99-2.47\frac{a}{h}+12.97{\left(\frac{a}{h}\right)}^{2}-23.17{\left(\frac{a}{h}\right)}^{3}+24.80{\left(\frac{a}{h}\right)}^{4}$$

where P was the largest load before fracture, L was the supporting span, b and h were the length, width, and thickness for the testing bars, and a was the notch depth. Each value was determined by taking an average of 5 specimens.

The dynamic mechanical properties of the ZrO_2_ ceramics with different photosensitive resins were measured using a split Hopkinson pressure bar (SHPB, Henan Lamp Electromechanical Technology Co., Ltd., Luoyang, Henan, China). The dynamic stress–strain curves were obtained according to the load and displacement of the pressure bar.

## 3. Results and Discussion

### 3.1. Printing Performance

**Figure 3:**
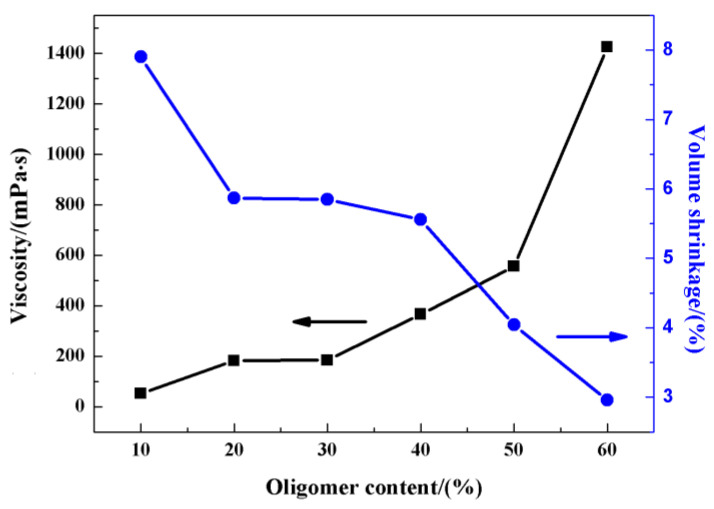
The viscosities and volume shrinkage of photosensitive resins with different contents of oligomers.

The viscosity and volume shrinkage of photosensitive resin as a function of the oligomer content are displayed in Figure 3. With increasing content of PUA, the photosensitive resin exhibited increasing viscosity. When the PUA content increased from 10% to 50%, the viscosity of the photosensitive resin increased slowly from 52 mPa·s to 550 mPa·s. However, as the PUA content increased from 50% to 60%, the viscosity showed a sharp increase to 1425 mPa·s. The molecular weight of PUA is up to 15,000~20,000, and the molecular weight of monomers is smaller than 300; therefore, the increased content of PUA resulted in higher viscosity.

According to Figure 3, the volume shrinkage of photosensitive resin decreases with increasing content of PUA. The volume shrinkage shows a maximum of 7.9% when the PUA content is 10% and reaches a minimum of 2.96% with 60% PUA. The essence of the photopolymerization process is that the photoinitiator triggers the polymerization of small molecules into large molecules. In the process of polymerization, the molecular bond is converted from the van der Waals bond into a covalent bond, thereby causing volume shrinkage after curing. With the increased proportion of oligomer in the photosensitive resin system, the overall reaction degree was getting lower, decreasing the concentration of double bonds and degree of crosslinking in the resin, which further led to a decreasing trend in the volume shrinkage rate of the cured product.

**Figure 4:**
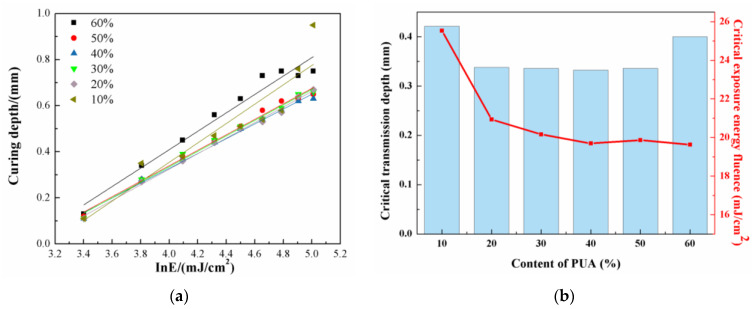
(**a**) The curing depth plotted versus ln*E* along with the linear fitting; (**b**) the critical transmission depth and critical exposure energy fluence of the photosensitive resins with different contents of PUA.

As shown in Table 1, the curing depth (*C_d_*) of photosensitive resins becomes larger with increasing exposure energy fluence (*E*). In Figure 4a, *C_d_* is plotted versus ln*E.* The critical exposure energy fluence and critical transmission depth are obtained based on the intercept and slope of the fitting line, and plotted as a function of PUA content in Figure 4b; the photosensitive resins show large critical transmission depth of above 0.4 mm as the contents of PUA are 10% and 60%, and the other resins exhibit a critical transmission depth of merely 0.33 mm. The critical exposure energy fluence exhibits a decrease with increasing content of PUA in the photosensitive resin, and the value of critical exposure energy fluence is 25.5 mJ/cm^2^ with 10% PUA, which further decreases to below 20 mJ/cm^2^ as the content of PUA is no less than 40%. Generally speaking, the smaller critical exposure energy fluence makes it easier for the photosensitive resin to transform into bulks by UV curing under a certain energy [34] Therefore, 40%~60% PUA promotes the curing of photosensitive resin, which can be beneficial to the properties of 3D-printed samples.

When the content of PUA is 60%, the photosensitive resin shows the lowest critical exposure and volume shrinkage rate; however, its high viscosity hinders the flowability of the ceramic slurry and reduces the molding accuracy, which is unwanted in 3D printing. As the content of PUA is lower than 40%, the low viscosity of the photosensitive resin is beneficial to the flowability of the ceramic slurry, but the large volume shrinkage results in excess pores and uncontrollable dimensional change. In comparison, the photosensitive resins with 50% PUA show small volume shrinkage, media viscosity, and low critical exposure, which is conducive to the operation and curing. Therefore, the photosensitive resin with 50% PUA is regarded as an optimized formula for subsequent 3D printing of ZrO_2_ ceramics.

### 3.2. Microstructure of the Ceramics

**Figure 5:**
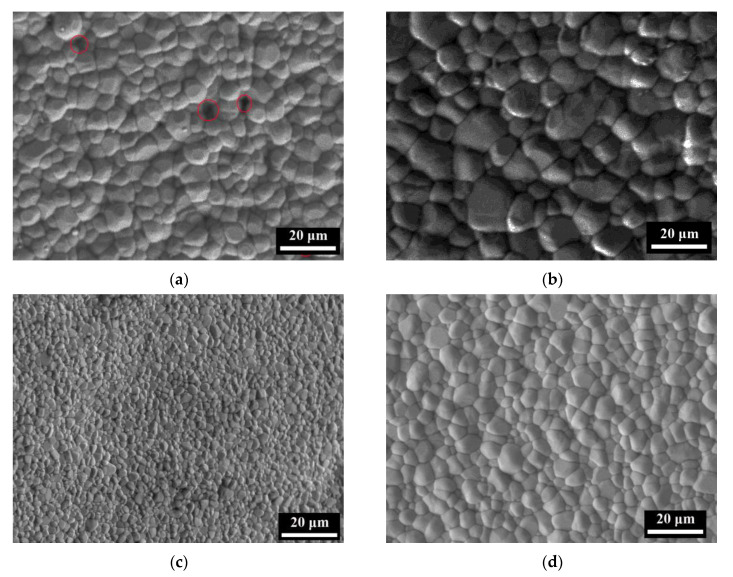
SEM images of the 3D-printed ceramics after: (**a**) CS at 1500 °C; (**b**) CS at 1600 °C; (**c**) MS at 1500 °C; (**d**) MS at 1600 °C, and the red circles denote the pores.

The ceramics prepared by 3D printing with 50% PUA were sintered through conventional sintering and microwave sintering at 1500 °C and 1600 °C, and the SEM images are displayed in Figure 5. According to Figure 5a, a small number of pores are detected in the sample sintered by CS at 1500 °C, as marked by red circles in the SEM, and the average grain size is about 5.1 μm. As the CS temperature increases to 1600 °C, most of the pores disappear but the grain size increases significantly with an average value of 10.9 μm. In comparison, the sample shows a dense microstructure and fine grain sizes after MS. Almost no pores are detected after MS at 1500 °C and 1500 °C, and the mean grain size is about 2.1 μm and 3.9 μm, respectively.

**Figure 6:**
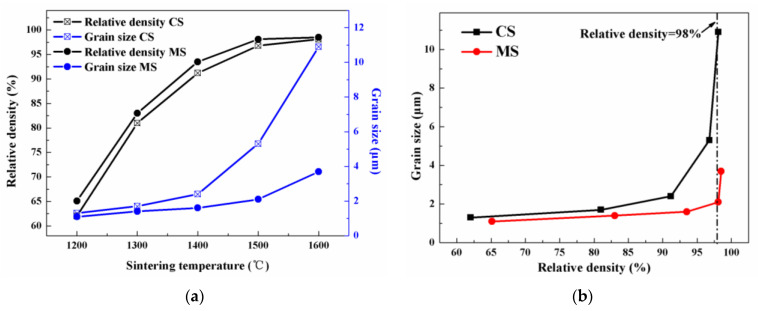
(**a**) The relative density and grain size of ceramics after CS and MS at different temperatures; (**b**) the grain size as function of relative density after CS and MS.

The relative density and average grain size of ceramics with 50% PUA after CS and MS at 1200~1600 °C are tested and plotted versus sintering temperature in Figure 6a. The trajectories of relative density versus sintering temperature are similar to each other, where the increase in relative density is quite sharp at low temperatures and becomes gentle at high temperatures. At the given temperature, the relative density after MS is slightly higher than the value after CS. The grain size shows a J-shaped growth with increasing temperature, and the severe grain growth at high temperature is similar to the sintering behavior of various ceramics. Furthermore, the grain size in MS is significantly smaller than that in CS. As the temperature increases from 1200 °C to 1600 °C, the grain size increased from 1.1 μm to 3.9 μm in MS, but 1.2 μm to 10.9 μm in CS. Therefore, the severe grain growth is suppressed by MS based on the rapid heating. According to previous works [32,33], microwave sintering is beneficial to the densification of ionic conductors due to the high coupling via ionic mobility. However, the temperature homogeneity is difficult to be controlled and the grain size can be uneven. In this work, the excessive growth of ZrO_2_ particles is invisible after microwave sintering, and uniform distribution of grain size is beneficial to the mechanical property of ceramics.

The grain size of ceramics prepared by 3D printing with 50% PUA is plotted versus the relative density in Figure 6b, and the grain growth is significant in high relative densities compared to low densities. As the relative density is low, the grains are surrounded by a mass of pores, and the grain growth is mainly conducted by surface diffusion. In comparison, the grains are interconnected to each other as most of the pores had been removed with high relative densities, and the grain boundary shift becomes the primary reason for the growth of grains. To achieve relative density above 98%, the lowest sintering temperature should be 1600 °C in CS, and the average grain size is up to 10.9 μm; however, the threshold temperature in MS is 1500 °C, and the average grain size is merely 2.1 μm. Compared to CS, MS was beneficial to dense microstructure with fine grain size. After MS at 1400 °C, the relative density is merely 93.5%. The sample MS at 1500 °C shows a high relative density of 98.3% and fine grain size of 2.1 μm. As the temperature is 1600 °C in MS, the relative density is slightly higher of 98.5%, and the grain size increases to about 4 μm. Generally speaking, high relative density and small grain size are beneficial to the mechanical strength of armor ceramics. Therefore, MS 1500 °C was confirmed as an optimized sintering process for the 3D-printed ZrO_2_ ceramics.

**Figure 7:**
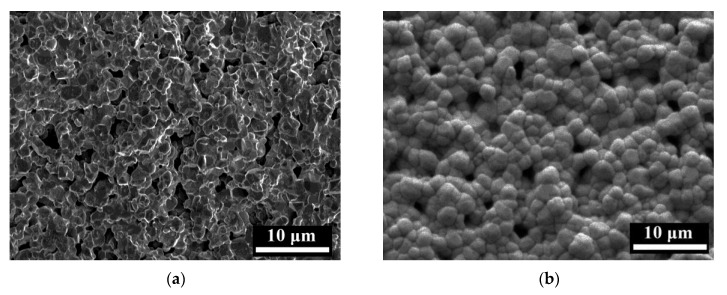

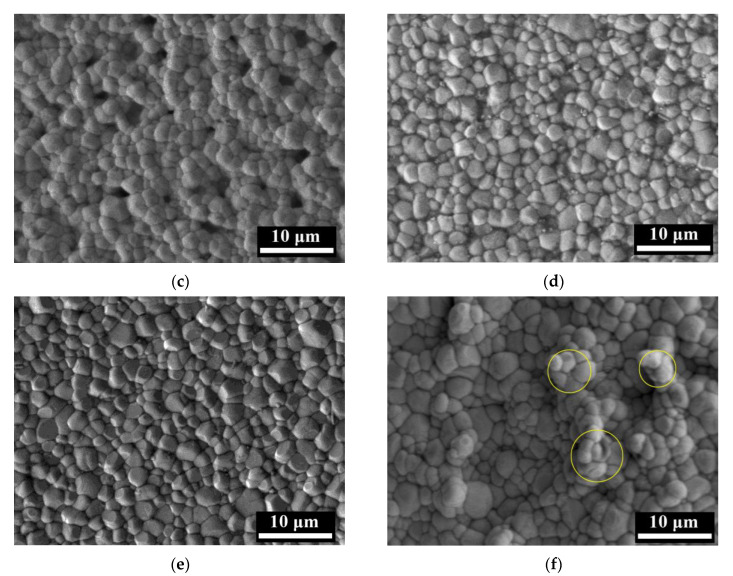
SEM images of the 3D-printed ceramics with different contents of PUA: (**a**) 10%; (**b**) 20%; (**c**) 30%; (**d**) 40%; (**e**) 50%; (**f**) 60% after MS at 1500 °C, and the yellow circles denote the aggregation.

The samples 3D-printed with different contents of PUA were sintered by MS at 1500 °C, and the SEM images are shown in Figure 7. With 10% PUA in the photosensitive resin, there are quantities of interconnected pores in the SEM image in Figure 7a, attributed to the low viscosity and large volume shrinkage according to Figure 3. As the content of PUA in photosensitive resins increases to 20%~40%, the viscosity increases and the volume shrinkage decreases significantly; therefore, the samples with 20%~40% PUA show larger relative densities, and most of the pores developed into discrete pores in the SEM images in Figure 7b–d. As the PUA content in photosensitive resin is 50%, the ceramics show dense and uniform with almost no detectable pores in Figure 7e. However, with 60% PUA in the photosensitive resin, the ceramic sample shows obvious particle agglomeration in the SEM image, as marked by yellow circles in Figure 7f, attributed to the high viscosity of photosensitive resin, and the particle agglomeration further leads to a rough surface and inhomogeneous microstructure with bits of pores around the agglomerates. The SEM images further confirm that the photosensitive resin with 50% PUA is an optimized formula for 3D printing of ZrO_2_ ceramics.

### 3.3. Mechanical Property

**Figure 8:**
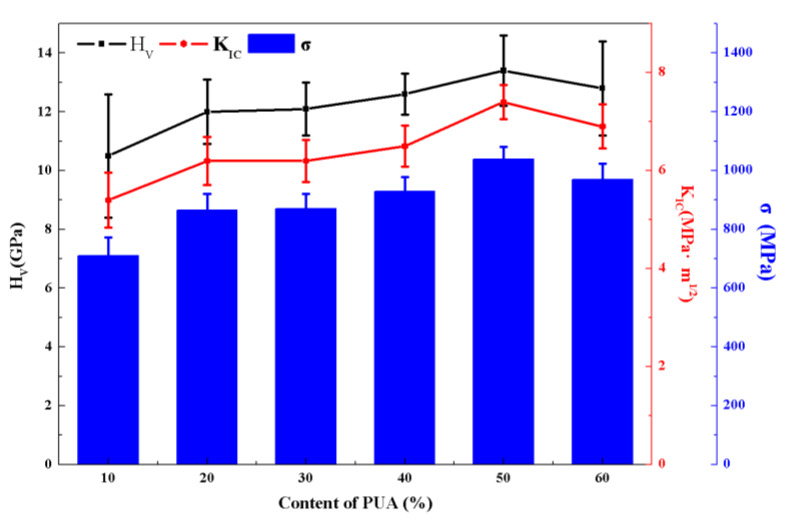
Vickers hardness (H_V_), fracture toughness (K_IC_), and flexure strength (σ) of the 3D-printed ceramics with different contents of PUA after MS at 1500 °C.

Mechanical properties, i.e., Vickers hardness (H_V_), fracture toughness (K_IC_), and flexure strength (σ) of the 3D-printed ceramics with different monomers after MS at 1500 °C, were measured and plotted in Figure 8. The mechanical properties are highly dependent on the microstructures in Figure 7. With increasing PUA from 10% to 50% in the photosensitive resin, the mechanical properties of the ceramics are better and better, which is attributed to the elimination of residual pores. As the content of PUA further increases to 60%, the mechanical properties of the ceramics are degraded, resulting from the agglomerates and pores in the microstructure. The sample with 50% PUA in the photosensitive resin exhibits superior mechanical properties, including a Vickers hardness of 13.4 GPa, fracture toughness of 7.4 MPa·m^1/2^, and flexure strength of 1038 MPa, which was by virtue of the dense, uniform microstructure with fine grains.

**Figure 9:**
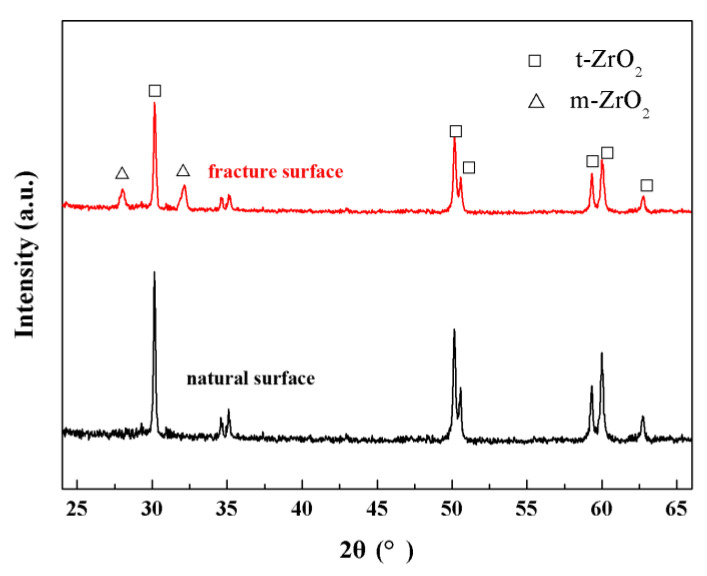
XRD patterns recorded from the natural surface and fracture surface of the ZrO_2_ ceramics after MS at 1500 °C.

XRD patterns were recorded from the natural surface and fracture surface of the ZrO_2_ ceramics after MS at 1500 °C, as shown in Figure 9. The natural surface showed only characteristic peaks for t-ZrO_2_ (PDF#79-1768) before fracture, without any visible peaks for m-ZrO_2_ in the XRD patterns, which is attributed to the stabilization of 3 mol% Y_2_O_3_. After fracture, the sample showed small quantities of m-ZrO_2_ (PDF#37-1487) in the fracture surface, confirming phase transformation from t-ZrO_2_ to m-ZrO_2_ in the process of fracture, which is highly beneficial to the mechanical property of the ZrO_2_ ceramics.

**Figure 10:**
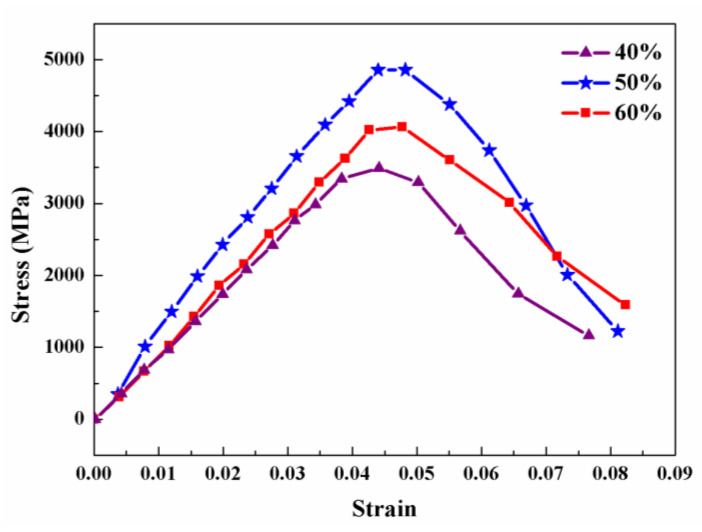
Dynamic stress–strain curves of the alumina ceramics 3D-printed with different contents of PUA after MS at 1500 °C.

The dynamic mechanical properties of the ZrO_2_ ceramics after MS at 1500 °C were evaluated through an SHPB test with a strain rate of 1200 s^−1^, and the dynamic stress–strain curves are displayed in Figure 10. Each of the ZrO_2_ ceramics displays an elastic deformation larger than 4%. After the dynamic stress reaches the maximum value, a nonlinear strain is shown, due to the main crack expansion under high strain rates. Even though the largest strain before fracture is as high as 6%, this is a typical feature of brittle ceramics, rather than plastic deformation. As is different from the static mechanical measurements, the largest stress and strain in dynamic tests are much higher. In SHPB tests, the loading rate is quicker than the spreading of cracks; therefore, the crack spreading is delayed and the fracture is tardy, making the strength and strain values larger at dynamic mechanical tests. Compared to the other samples, the ZrO_2_ ceramics with 50% PUA display the highest dynamic strength of 4.9 GPa, due to the uniform and fine-grained microstructure with high relative density. Similar to the static flexure strength, the dynamic strength is also confirmed to be affected by the residual pores and agglomerates in the ceramics.

## 4. Conclusions

ZrO_2_ armor ceramics were prepared by 3D printing with different contents of PUA along with microwave sintering. The appropriate formula of photosensitive resin was investigated via the 3D printing and the optimal sintering method was determined through the grain size and porosity after sintering. Furthermore, the effects of PUA content on the microstructures and mechanical properties of 3D-printed alumina ceramics were investigated:

(1) The photosensitive resin with 50% PUA is confirmed as an optimized formula for subsequent 3D printing of ZrO_2_ ceramics, as the photosensitive resin shows a small volume shrinkage of 4.05%, media viscosity of 550 mPa·s, and low critical exposure of 20 mJ/cm^2^, which is conducive to the operation and curing;

(2) To achieve relative density of the ZrO_2_ ceramics above 98%, the sintering temperature is 1600 °C in CS, and the average grain size is 10.9 μm; however, the threshold temperature in MS is 1500 °C, and the grain size is merely 2.1 μm. Compared to CS, MS is beneficial to a dense microstructure with fine grain size, and MS 1500 °C is confirmed as an optimized sintering procedure for the 3D-printed ZrO_2_ ceramics;

(3) The PUA content further affects the microstructure and mechanical property of the 3D-printed ZrO_2_ ceramics. The samples with 10%~40% PUA show some pores due to the low viscosity and large volume shrinkage of photosensitive resins, and the sample with 60% PUA exhibits agglomeration and an inhomogeneous microstructure with pores. Finally, the ZrO_2_ ceramics by 3D printing with 50% PUA show excellent mechanical properties, with a Vickers hardness of 3.4 GPa, fracture toughness of 7.4 MPa·m^1/2^, flexure strength of 1038 MPa, and dynamic strength at 1200 s^−1^ of 4.9 GPa. This work provides guidance for high-performance ZrO_2_ armor ceramics by 3D printing and microwave sintering.

## Figures and Tables

**Table 1:** Curing depth (*C_d_*, mm) of photosensitive resin with different contents of PUA under different exposure energy fluences (*E*, mJ/cm^2^).

*E*	30	45	60	75	90	105	120	135	150
*C_d_*									
PUA Content									
10%	0.11	0.35	0.38	0.47	0.51	0.54	0.58	0.76	0.95
20%	0.11	0.27	0.36	0.44	0.50	0.53	0.57	0.64	0.67
30%	0.11	0.28	0.39	0.45	0.51	0.54	0.59	0.65	0.66
40%	0.11	0.28	0.37	0.44	0.50	0.54	0.58	0.62	0.63
50%	0.12	0.28	0.38	0.45	0.51	0.58	0.62	0.63	0.65
60%	0.13	0.34	0.45	0.56	0.63	0.73	0.75	0.73	0.75

## Data Availability

The original contributions presented in this study are included in the article. Further inquiries can be directed to the corresponding authors.
